# Relieving effect of an Maren-Zhizhu emulsion on loperamide hydrochloride-induced constipation in mice and its effects on the gut microbiota

**DOI:** 10.3389/fmicb.2025.1641367

**Published:** 2025-08-13

**Authors:** Xinran Han, Yuankun Chai, Na Li, Chunyu Li, Fan Yao, Qiang Huang, Lili Weng, Zhidong Qiu, Ailing Jia

**Affiliations:** ^1^College of Pharmacy, Changchun University of Chinese Medicine, Changchun, China; ^2^Siping Central People’s Hospital, Siping, China

**Keywords:** traditional Chinese medicine, constipation, emulsion, transcriptomics, gut microbiota

## Abstract

**Introduction:**

Constipation, a common gastrointestinal disorder, is rapidly increasing in prevalence worldwide. An increasing number of individuals are choosing traditional Chinese medicine (TCM) as an adjunctive treatment for constipation. In this study, the effect of Maren-Zhizhu emulsion (MRZZ) prepared with *Cannabis sativa* L. (Huomaren), *Rehmannia glutinosa* Libosch. (Dihuang), *Citrus aurantium* L. (Zhiqiao), *Atractylodes macrocephala* Koidz. (Baizhu), and tiger nut oils from *Cyperus esculentus* L. on relieving loperamide hydrochloride-induced constipation in mice was evaluated.

**Methods:**

MRZZ was administered orally at low (0.65 g/kg) and high (2.6 g/kg) doses for 14 consecutive days. Loperamide hydrochloride (4 mg/kg) was used to induce constipation in male ICR mice. Colon tissue pathology, transcriptomics, and changes in the gut microbiota were analyzed to assess the efficacy of MRZZ in alleviating constipation.

**Results and discussion:**

We observed changes in fecal water content, time of first black stool, gastrointestinal transit rate, short-chain fatty acid (SCFAs) levels, and serum gastrointestinal regulatory peptide levels before and after consuming MRZZ. MRZZ increased the levels of relevant gastrointestinal regulatory neurotransmitters such as MTL, SP, and GAS, as well as SCFAs (especially acetate and isobutyrate). Furthermore, it reshaped the structure of the gut microbiota by increasing the relative abundance of *Firmicutes* and reducing potentially pathogenic bacteria, such as *Proteobacteria.* In addition, MRZZ suppressed intestinal inflammatory responses and enhances intestinal functions. In conclusion, MRZZ may alleviate constipation by synergistically regulating the gut microbiota, which may enhance the application value of TCM to treat constipation.

## 1 Introduction

Constipation is a common gastrointestinal disorder (Barberio et al., 2021). Symptoms include a decreased frequency of bowel movements and incomplete bowel movements (Bharucha and Lacy, 2020). Constipation reduces patients’ quality of life and may lead to mental health issues such as anxiety and depression, with its prevalence steadily rising due to factors such as excessive stress and irregular dietary habits (Kirindage et al., 2024; Mugie et al., 2011). Approximately 10.1–15.3% of adults and 9.5% of children worldwide regularly experience constipation (Vos et al., 2024; Zhu et al., 2024), and the incidence rate has been positively correlated with increasing age (Bisht et al., 2023). These findings indicate that constipation should be urgently addressed.

The gut microbiota is a complex ecosystem composed of bacteria, archaea, viruses, fungi, and eukaryotic microorganisms such as protozoa (Illiano et al., 2020). Constipation is closely related to the gut microbiota, the alteration of which is often accompanied by intestinal diseases like colitis and gastroenteritis, thus affecting intestinal peristalsis (Huang et al., 2018; Obata et al., 2020). The gut microbiota also affects the short-chain fatty acid (SCFA) and metabolite contents in the body (Wang et al., 2025). Changes in the number and type of microorganisms in feces can provide a reference for evaluating the effectiveness of constipation treatment (Dowgiałło-Gornowicz et al., 2024). Exogenous factors such as diet and medication can significantly affect the gut microbiota (Tian et al., 2022). Currently, laxatives and stool softeners are commonly used to treat constipation (Hojo et al., 2025). Although medication can quickly relieve constipation symptoms, it may cause side effects such as cardiovascular disease and intestinal nerve damage, and long-term use may lead to drug dependence (Bharucha et al., 2013; Sayuk et al., 2022). Therefore, an increasing number of individuals are choosing traditional Chinese medicine (TCM) as an adjunctive treatment for constipation (Wang et al., 2024).

*Cannabis sativa* L. (Huomaren) moisturizes the intestines and is rich in unsaturated fatty acids and nutrients, which can effectively promote intestinal peristalsis and relieve constipation (Li et al., 2022; Tănase Apetroaei et al., 2024). *Rehmannia glutinosa* Libosch. (Dihuang) is often used as a complementary drug to nourish Yin and reinforce the kidney, and its quercetin can effectively protect the barrier function of the intestinal mucosa and alleviate the symptom of defecation difficulty (Liu and Zhi, 2021; Xu et al., 2022). *Cannabis sativa* L. and *Rehmannia glutinosa* Libosch. are medicinal and food ingredients widely used in food processing. *Aurantii Fructus* from *Citrus aurantium* L. (Zhiqiao) regulates qi flow to activate stagnancy, and its flavonoid components can influence the composition of the intestinal microbiota and promote gastric emptying and small intestinal propulsion in mice (Stevens et al., 2019). In addition, *Atractylodes macrocephala* Koidz. (Baizhu) can invigorate the spleen for diuresis, regulate the water balance in the intestinal tract, and reduce the symptoms of constipation (Qin et al., 2024). Tiger nut oil from *Cyperus esculentus* L., which is rich in unsaturated fatty acids, phospholipids, and other beneficial components, exhibits a mild effect with rapid body absorption and supports intestinal lubrication, making it suitable for long-term consumption (Sánchez-Zapata et al., 2012; Zhang and Sun, 2023). However, there are no reports on the laxative effects of the above four Chinese herbs combined with tiger nut oil.

Therefore, to reduce the limitations of traditional laxatives and improve the application of TCM in constipation, we developed the health food product Maren-Zhizhu emulsion (MRZZ)—comprising *Cannabis sativa* L., *Rehmannia glutinosa* Libosch., *Citrus aurantium* L., *Atractylodes macrocephala* Koidz., and tiger nut oils. We hypothesized that MRZZ could relieve loperamide hydrochloride-induced constipation. We used male ICR mice to model loperamide hydrochloride-induced constipation and studied the effect of MRZZ on constipation relief, focusing on the gut microbiota and other factors. Chinese herbs have been incorporated into food products to enhance the comprehensive application value of TCM. As an emulsion, MRZZ’s microemulsion system of MRZZ can increase the contact area between the intestinal tract and nutrients, enhance the bioavailability and absorption time of active ingredients, and synergistically enhance the absorption efficiency of medicinal substances (Borreani et al., 2019), meeting the needs of the functional food market in the emulsion field.

## 2 Materials and methods

### 2.1 Materials

*Cannabis sativa* L., *Rehmannia glutinosa* Libosch., *Citrus aurantium* L., and *Atractylodes macrocephala* Koidz. were purchased from the Jilin Beiyao Pharmaceutical Group Co., Ltd. (Jilin, China) and identified by Professor Lili Weng at the College of Pharmacy, Changchun University of Chinese Medicine. Tiger nut oil was purchased from Kunhua Biotechnology Co., Ltd. (Henan, China). Chocolate flavoring (PR-1573) was purchased from Hasegawa Flavors and Fragrances Co., Ltd. (Shanghai, China), and crystalline fructose was purchased from Shandong Xiwang Sugar Industry Co., Ltd. (Shandong, China), and fructose syrup was purchased from Shandong Xiangchi Jianyuan Biotechnology Co., Ltd. (Shandong, China). Potassium sorbate was purchased from Nantong Aokai Biotechnology Development Co., Ltd. (Jiangsu, China), and phospholipids from Anhui Yuanchuang Technology Co., Ltd. (Anhui, China), and xanthan gum from Ordos Zhongxuan Biochemical Co. Ltd. (Inner Mongolia Autonomous Region, China). Enzyme-linked immunosorbent assay (ELISA) kits for substance P (SP), gastrin (GAS), and motilin (MTL) were purchased from Jiangsu Meimian Industrial Co. Ltd. (Jiangsu, China). Loperamide hydrochloride powder (CAS number 34552–83-5, strength ≥ 98%) was purchased from Shanghai Yuanye Biotechnology Co. Ltd. (Shanghai, China). Xiangdanqing capsules (0.4 g per capsule) were purchased from Nianqing Bao Pharmaceutical Co. Ltd. (Shanxi, China).

### 2.2 Preparing the materials

The MRZZ formula is based on prescriptions developed by clinical doctors at the Affiliated Hospital of the Changchun University of Chinese Medicine. To prepare MRZZ, we decocted *Cannabis sativa* L.*, Rehmannia glutinosa* Libosch., *Citrus aurantium* L., and *Atractidurides macrocephala* Koidz. were decocted in a ratio of 3:5:6:6 twice with a certain amount of water under normal pressure conditions, boiling at 100°C and then reducing the heat to low and starting the timer, for 1.5 h each time. The amount of water used for the two decoctions was 10 and 8 times the weight of the herbs, respectively. The decoctions were filtered, the filtrates were combined, and the resulting supernatant was collected via centrifugation at 8000 rpm for 20 min and concentrated to 1 g/mL. The concentrate was added to crystallized fructose, fructose syrup, and potassium sorbate in the aqueous phase. Then, 50% tiger nut oil, phospholipids, and chocolate flavoring were added to the mixture, which was heated until the added contents dissolved. The oil phase was shear-mixed at 8000 rpm for 2 min. The aqueous phase was added to the oil phase while continuing shear mixing at 8000 rpm, and xanthan gum was added to obtain colostrum. The colostrum was passed through a high-pressure homogenizer (400 bar with three cycles). Filling and sterilization processes yielded the final product. MRZZ is an oil-in-water (O/W) emulsion with an average particle size of 544.6 nm.

For the activated carbon solution preparation, we accurately weighed 100 g of gum arabic, added 800 mL of water, and boiled the solution until it became transparent. Next, 50 g of powdered activated carbon was added to the above solution, which was boiled three times, cooled, diluted to 1,000 mL with water, refrigerated at 4°C, and shaken well before use.

### 2.3 Animal model and sample collection

Fifty healthy six-week-old male ICR mice (specific pathogen-free; 20–22 g) were purchased from Liaoning Changsheng Biotechnology Co., Ltd. (animal certificate number SCXK[Liao]2020–0001). The mice were acclimatized and fed for 1 week under barrier environment laboratory conditions (room temperature: 23 ± 2°C, relative humidity: 50% ± 10%, 12 h light/dark cycle). Food and water were provided *ad. libitum*.

When acclimatization ended, the mice were randomly divided into five groups (n = 10): normal control (NC), model control (MC), positive drug (PD), low-dose (LD), and high-dose (HD) groups. The PD group was gavaged with Xiangdanqing capsules (0.208 g/kg) once daily, whereas the LD and HD groups were gavaged with the MRZZ, with herbal dosages of 0.65 and 2.6 g/kg, respectively, once daily for 14 days. During this period, the NC and MC groups were administered 0.2 mL of saline solution. On days 15–17, except for the NC group, which was administered saline solution, the other groups were administered oral loperamide hydrochloride (4 mg/kg) daily for 3 consecutive days to establish a constipation model in mice. During this period, the PD, LD, and HD groups were administered MRZZ and Xiangdanqing capsules. After fecal sampling, blood was collected via retro-orbital bleeding, and the mice were decapitated and sacrificed. Tissue specimens were then collected.

### 2.4 Evaluating the defecation function

Weight changes in mice were recorded and monitored daily. On day 17 after the last administration, the mice were transferred to individual cages and fecal samples were collected within 3 h. The wet weight of the fresh feces was then recorded. All mice were fasted for 16 h without water restriction. Except for the NC group, which was administered saline, all other mice were administered 4 mg/kg of loperamide hydrochloride. After 30 min, all mice were orally administered 0.2 mL of activated carbon solution, and the time from the completion of gavage to the first defecation of the black feces was recorded. Fecal water content was calculated using the following equation: fecal water content (%) = ([wet weight − dry weight]/wet weight) × 100%.

### 2.5 Small intestine propulsion testing

The gastrointestinal transit rate was assessed using the method of the first black stool test in mice. Thirty minutes after the last dose, 0.2 mL of activated carbon solution was administered via gavage. After 25 min, the mice were euthanized via cervical dislocation. The intact small intestine was dissected, and the distance from the pylorus to the end of the activated carbon solution was measured as the migration distance. The following equation was used to calculate the small intestinal transit rate (%): small intestinal transit rate (%) = (transit distance of the activated carbon/total length of the small intestine) × 100%.

### 2.6 Hematoxylin and eosin (H&E) staining of small intestine tissue

Following the method described by Li et al. (2022), mice were decapitated and sacrificed, and the small intestine was dissected from the cecum to the anterior end of the anus and fixed with 4% paraformaldehyde. All samples were embedded in paraffin and sectioned to a thickness of 5 μm. The sections were deparaffinized in xylene, rehydrated sequentially with alcohol and distilled water, and stained with H&E. A microscope (Eclipse CI, Nikon Corporation, Japan) was used to observe the histological differences in the colon.

### 2.7 Immunofluorescence analysis of small intestine tissue

Following the method described by Li et al. (2022), paraffin sections were deparaffinized, rehydrated in xylene, and antigenically repaired using a citric acid antigen repair buffer (pH = 6). After incubation pretreatment with 10% goat serum, zonula occludens-1 (ZO-1) and occludin (prepared at a dilution ratio of 1:200 in phosphate-buffered saline [PBS; pH = 7.4]) were added dropwise, and the sections were incubated at 4°C overnight. After washing with PBS, the sections were immersed in a solution containing a secondary Alexa 488-conjugated goat anti-mouse antibody and incubated at room temperature for 50 min. The nuclei were re-stained with drops of 4′,6-diamidino-2-phenylindole (DAPI) staining solution before sealing the slices with a fluorescence-quenching sealer. Sections were observed under a fluorescence microscope (Eclipse CI, Nikon Corporation, Japan), and images were captured at 400 × magnification to observe inflammatory infiltration and tissue damage.

### 2.8 Serum gastrointestinal regulatory peptides in mice

Blood was collected via retro-orbital bleeding of mice and left for 15–30 min before centrifuging at 3000 rpm for 15 min at 4°C. The supernatant (serum) was separated and frozen at −80°C for further biochemical analysis. SP, MTL, and GAS levels in the serum were detected using commercial ELISA kits according to the manufacturer’s instructions. Absorbance was measured at 450 nm using an enzyme marker (SER33270-1236, Molecular Devices, Shanghai, China).

### 2.9 Determining fecal SCFA contents

The SCFA content in mice feces was determined using gas chromatography–mass spectrometry (GC–MS; Huang et al., 2020). Briefly, 50 mg of lyophilized feces was mixed with 500 μL of water, homogenized, and centrifuged (12,000 rpm and 4°C for 10 min). Then, 200 μL of the resulting supernatant was mixed with 100 μL of 15% phosphoric acid, 20 μL of internal standard solution (4-methylpentanoic acid, 375 μg/mL), and 280 μL ether. The mixture was then centrifuged at 4°C and 12,000 rpm for 10 min, and the supernatant was collected. The supernatant was analyzed using a GC system (Thermo Trace 1,300, Thermo Fisher Scientific, United States) equipped with an Agilent HP-INNOWAX capillary column (30 m × 0.25 mm inside diameter × 0.25 μm) and a Thermo ISQ 7000 mass spectrometer (Thermo Fisher Scientific, United States).

### 2.10 Transcriptome analysis

The samples were shipped on dry ice to Personalbio (Shanghai, China), as described by Cockrum et al. (2020). Polyadenylated mRNA was enriched from total RNA using oligo (dT) magnetic beads, and the mRNA was fragmented to approximately 300 base pairs (bp) in length using ionic interruption. The first strand of complementary DNA was synthesized using RNA as a template, and the second strand was synthesized using a 6-base random primer and reverse transcriptase. After library construction, polymerase chain reaction (PCR) amplification was performed to enrich the library fragments, and fragments of approximately 450 bp were selected. Libraries containing different index sequences were then mixed proportionally according to the effective concentration of the libraries and the amount of data required. After mixing, they were uniformly diluted to 2 nM, and single-stranded libraries were generated through base denaturation. After purification, the libraries were subjected to paired-end next-generation sequencing using the Illumina sequencing platform.

### 2.11 Real-time quantitative PCR analysis

To validate the transcriptome sequencing data, five genes were selected for further analysis based on previously described methods (Cockrum et al., 2020). Glyceraldehyde-3-phosphate dehydrogenase (GAPDH) was used as a reference gene, and total RNA was extracted from the colon tissues using Invitrogen TRIzol reagent (15,596,018, Thermo Fisher Scientific, United States). The PrimeScript™ 1^st^ stand cDNA Synthesis Kit (TaKaRa Biotechnology Co., Ltd., Japan) was used to reverse transcribe the total RNA that had been tested and quantified into cDNA. Corresponding fluorescent quantitative PCR experiments were performed using the AceQ® qPCR SYBR® Green Master Kit (Vazyme Biotech Co., Ltd., Nanjing, China). For each target gene, the cDNA template of the sample was selected for the PCR reaction, which comprised an initial denaturation step of 5 min at 95°C, followed by 40 cycles of denaturation at 95°C for 15 s and annealing at 60°C for 30 s. Subsequent products were tested online using a real-time fluorescence quantifier (LightCycler480II, 384, Roche, Switzerland).

### 2.12 16S rRNA sequencing of gut microbiota

Following the method described by Zhang et al. (2024), nucleic acids were extracted using the MagBeads FastDNA Kit for Soil (116,564,384; MP Biomedicals, CA, United States) and subjected to 0.8% agarose gel electrophoresis to determine the molecular size (DYY-6C, Beijing Liuyi Biotechnology Co., Ltd., Beijing, China), DNA was quantified using a nanodrop device. Bacteria with amplicon lengths of approximately 480 bp (V3V4 region of the 16S rRNA gene) were PCR-amplified and sequenced using the Illumina platform (Illumina, San Diego, CA, United States) and specific primers 338F (5′-barcode+ACTCCTACGGGGAGGCAGCA-3′) and 806R (5’-GGACTACHVGGGTWTCTAAT-3′). Data processing (i.e., quality filtering, denoising, splicing, and chimera removal) was performed on the sequences using the DADA2 plug-in, and the data were analyzed using the GenesCloud platform.^1^

### 2.13 Data analysis

Statistical analyses were performed using Prism 10.4 (GraphPad Software, San Diego, CA, United States). One-way analysis of variance and Scheffe’s multiple-comparisons technique were used to assess the differences between groups. Statistical significance was set at *p* < 0.05. The correlation between the gut microbiota and the transcriptome was constructed using the Spearman algorithm to create a correlation matrix. The random matrix theory was used to determine the filtering threshold for the correlation values. The igraph package was then used to construct an association network, which was visualized using the R language and ggraph data package.

## 3 Results

### 3.1 Effect of MRZZ on body weight, defecation indicators, and the gastrointestinal transit rate in mice

**Figure 1:**
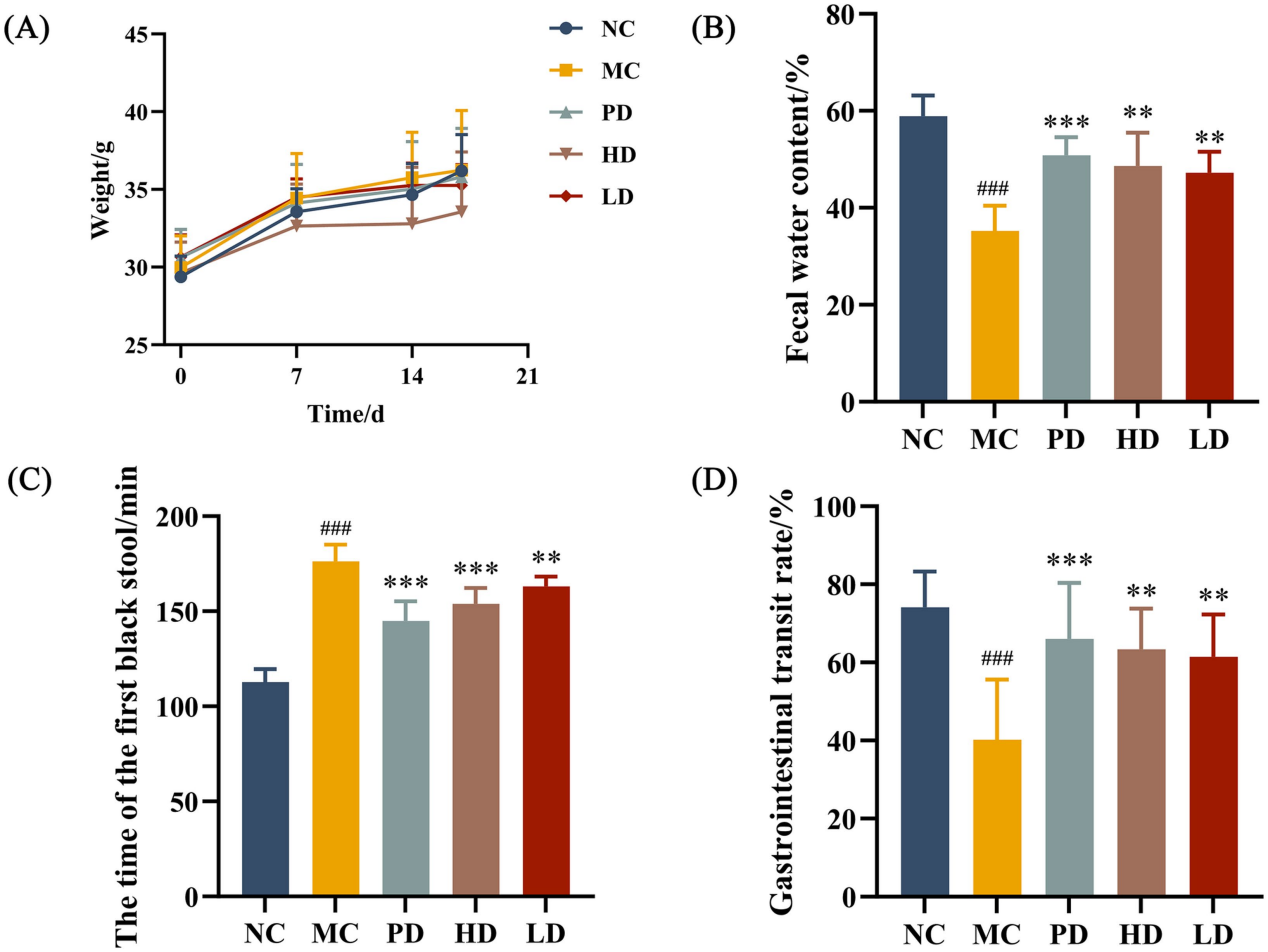
Effects of MRZZ on loperamide hydrochloride-induced constipation in mice. **(A)** Body weight. **(B)** Fecal water content. **(C)** Time to expulsion of the first black stools. **(D)** Small gastrointestinal transit rate. The data shown represent mean values (*n* = 10/group) and statistical significance: compared with the NC group: #*p* < 0.05, ##*p* < 0.01, ###*p* < 0.001; compared with the MC group: *p < 0.05, **p < 0.01, ***p < 0.001.

The body weights of the mice were recorded daily during the feeding period. The effects of MRZZ consumption on the general physiological status and fecal characteristics of the mice in each group were evaluated by monitoring body weight gain, fecal water content, time to the first black stool, and gastrointestinal transit rate. The MC group showed lower fecal water content, longer time to the first black stool passage, and lower small intestinal motility rate than the NC group (*p* < 0.01 in each case), suggesting that mice modeling of constipation was successfully generated (Figures 1A–D). After treatment, the differences between the PD, HD, and LD groups were significant compared to those of the MC group (*p* < 0.05), demonstrating that MRZZ significantly improved all indicators and alleviated constipation.

### 3.2 Histological H&E staining and immunofluorescence observation of the small intestine

**Figure 2:**
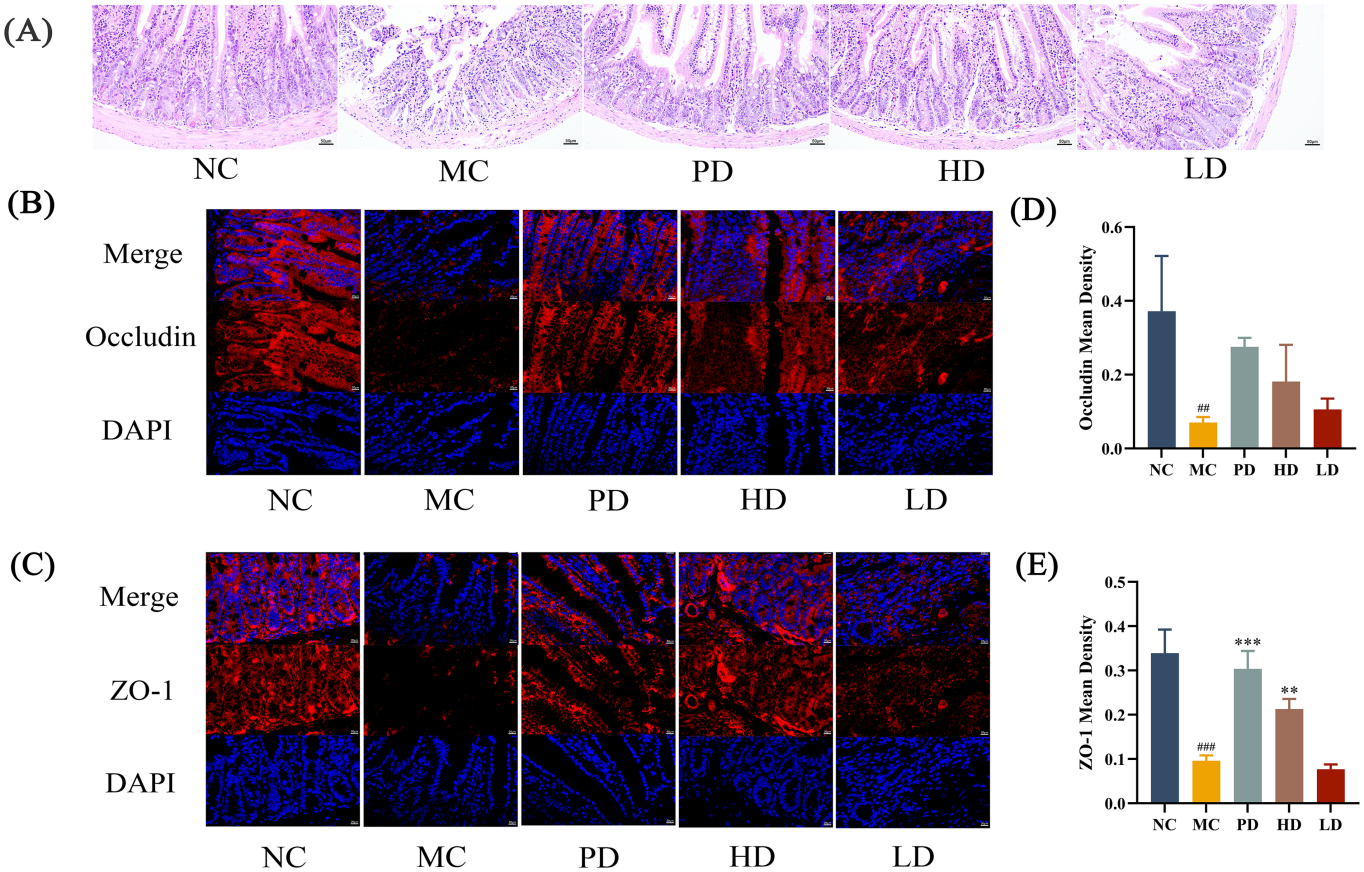
Morphology of the mice colon. **(A)** Hematoxylin and eosin (H&E) staining (200 × magnification). **(B)** Immunofluorescence staining with Occludin and DAPI (400 × magnification). **(C)** Immunofluorescence staining with ZO-1 and DAPI (400 × magnification). **(D)** Mean density of immunofluorescence staining for Occludin. **(E)** Mean density of immunofluorescence staining for ZO-1. Statistical significance: compared with the NC group: #*p* < 0.05, ##*p* < 0.01, ###*p* < 0.001; compared with the MC group: *p < 0.05, **p < 0.01, ***p < 0.001. Unlabeled datasets indicate no significant statistical difference between the NC group or MC group (*p* ≥ 0.05).

To further investigate the efficacy of MRZZ in alleviating constipation, histological staining was performed to detect changes in intestinal morphology. The intestinal tissue structure of the NC group mice was normal, whereas that of the MC group mice was markedly abnormal (Figure 2A). Disruption of intestinal villi in the field of view, extensive desquamation of epithelial cells in the mucosal layer, and a small number of inflammatory cells infiltrated the tissue. The intestinal villi structure was clear in the PD, HD, and LD groups, with neat and tight arrangements of epithelial cells in the mucosal layer, complete crypt structures, and no edema in the submucosal layer; their intestinal tissue structure was normal compared with that in the NC group.

After immunofluorescence staining, ZO-1 and Occludin appeared red, and the nuclei appeared blue after DAPI staining (Figures 2B,C). In the MC group, the fluorescence intensities of ZO-1 and Occludin were reduced, and obvious blue nuclei were visible in the DAPI plots. Compared with that in the MC group, the fluorescence intensity was increased in the PD and HD groups, and the mean density value of ZO-1 was significantly higher (*p* < 0.01; Figures 2D,E).

### 3.3 Effect of MRZZ on the serum contents of GAS, MTL, and SP in mice

**Figure 3:**
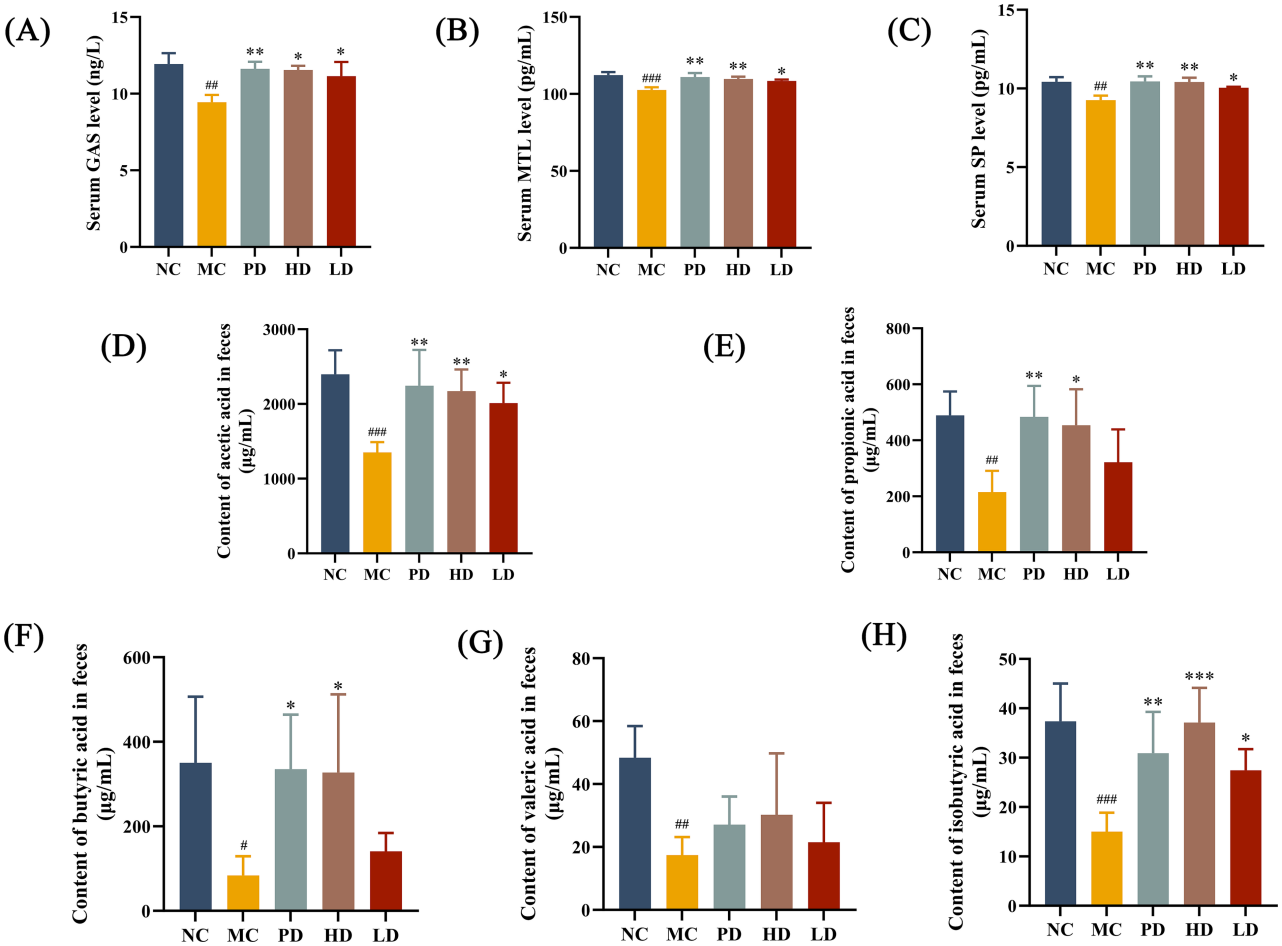
Determination of neurotransmitter activities in mice sera and short-chain fatty acids (SCFAs) in mice feces. **(A)** GAS. **(B)** MTL. **(C)** SP. The data shown represents the mean values. **(D)** Acetic acid. **(E)** Propionic acid. **(F)** Butyric acid. **(G)** Valeric acid. **(H)** Isobutyric acid. The data shown represent mean values (*n* = 5/group) and statistical significance: compared with the NC group: #*p* < 0.05, ##*p* < 0.01, ###*p* < 0.001; compared with the MC group: *p < 0.05, **p < 0.01, ***p < 0.001. Unlabeled datasets indicate no significant statistical difference between the NC group or MC group (*p* ≥ 0.05).

To verify the effects of MRZZ on the neurotransmitter content of mice with constipation, serum GAS, MTL, and SP levels were detected using ELISAs. The serum levels of GAS, MTL, and SP were significantly lower in the MC group than in the NC group (*p* < 0.05), and these indices increased after treatment with low and high doses of MRZZ and PD treatment (*p* < 0.05; Figures 3A–C). These data suggest that MRZZ significantly affects gastrointestinal regulatory peptide secretion in our mice model of loperamide hydrochloride-induced constipation.

### 3.4 Effect of MRZZ on SCFA contents in mice feces

SCFAs are mainly produced as metabolites by the gut microbiota and are closely related to the species and abundance of gut microbiota (Gao et al., 2021). Therefore, we used GC–MS to determine the SCFA content in mice feces. The fecal contents of acetic acid, propionic acid, butyric acid, valeric acid, and isobutyric acid in constipated mice were significantly decreased by loperamide hydrochloride treatment compared with those in the NC group (*p* < 0.05; Figures 3D–H). The acetic and isobutyric acid levels in the HD and LD groups were significantly higher than those in the MC group (*p* < 0.05). These results suggest that MRZZ can help maintain normal SCFA levels and restore intestinal function stability.

### 3.5 Transcriptomics analysis and RT-qPCR validation

**Figure 4:**
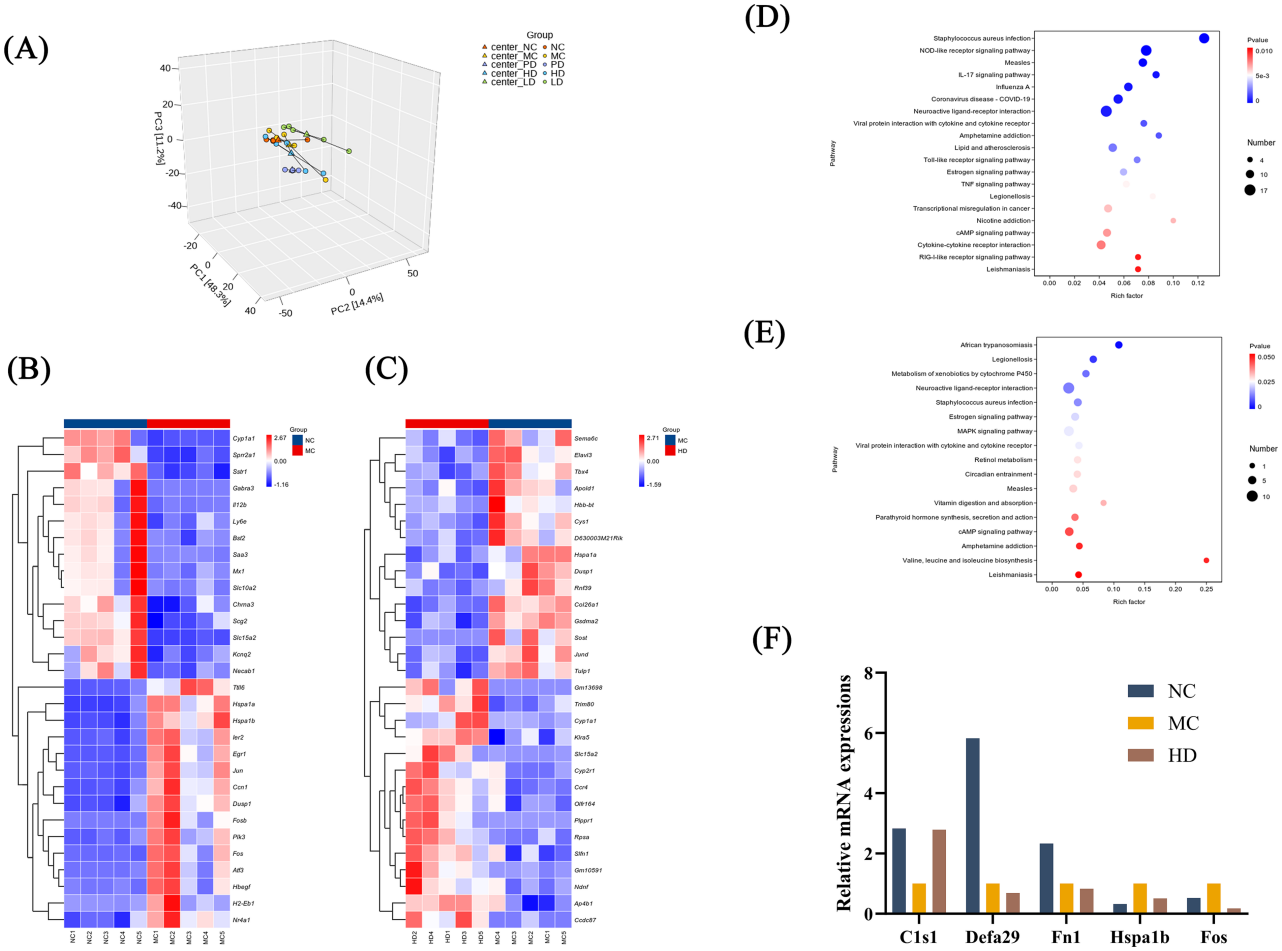
Analysis of mice fecal transcriptome data (*n* = 5). **(A)** Principal component analysis. **(B)** Top 20 differentially expressed genes between the NC and MC groups. **(C)** Top 20 differentially expressed genes in the MC and HD groups. **(D)** Factor plot of KEGG enrichment analysis for the NC and MC groups. **(E)** Factor plot of KEGG enrichment analysis for the MC and HD groups. **(F)** RT-qPCR-based validation of gene dysregulation observed in transcriptome data.

Differences in colonic gene expression patterns between the two groups (NC vs. MC and MC vs. HD) were investigated using transcriptome analysis. Principal component analysis combined with correlation analysis between the samples showed some reproducibility within the NC, MC, and HD groups, as well as significant differences between groups (Figure 4A).

Among the top 20 differentially expressed genes in the NC and MC groups, genes such as *Slc15a2, Cyp1a1, Sprr2a1*, and *Sstr1* in the MC group exhibited low expression, whereas genes such as *Egr1, Hspa1b, Jun, Hspa1a*, and *Dusp1* exhibited high expression (Figure 4B). Among the differentially expressed genes between the MC and HD groups, genes such as *Hspa1a, Dusp1, JunD,* and *Gsdma2* in the HD group showed low expression, whereas genes such as *Cyp1a1, Slc15a2, Ccr4,* and *Slfn1* showed high expression (Figure 4C). Kyoto Encyclopedia of Genes and Genomes (KEGG) analysis revealed that these differentially expressed genes were mainly enriched in pathways such as the NOD-like receptor signaling pathway, metabolism of xenobiotics by cytochrome P450, IL-17 signaling pathway, and estrogen signaling pathway (Figures 4D,E).

To verify the gene expression changes detected by RNA sequencing, five genes (*Hspa1b, Fos, C1s1, Defa29*, and *Fn1*) were randomly selected for RT-qPCR analysis (Figure 4F). The results were similar to those obtained by RNA sequencing. We hypothesized that MRZZ alleviates constipation in mice by regulating the expression of differentially expressed genes. Related pathways may play a key role in alleviating loperamide hydrochloride-induced constipation.

### 3.6 Effects of MRZZ on the *α*- and *β* diversity of the gut microbiota in mice

**Figure 5:**
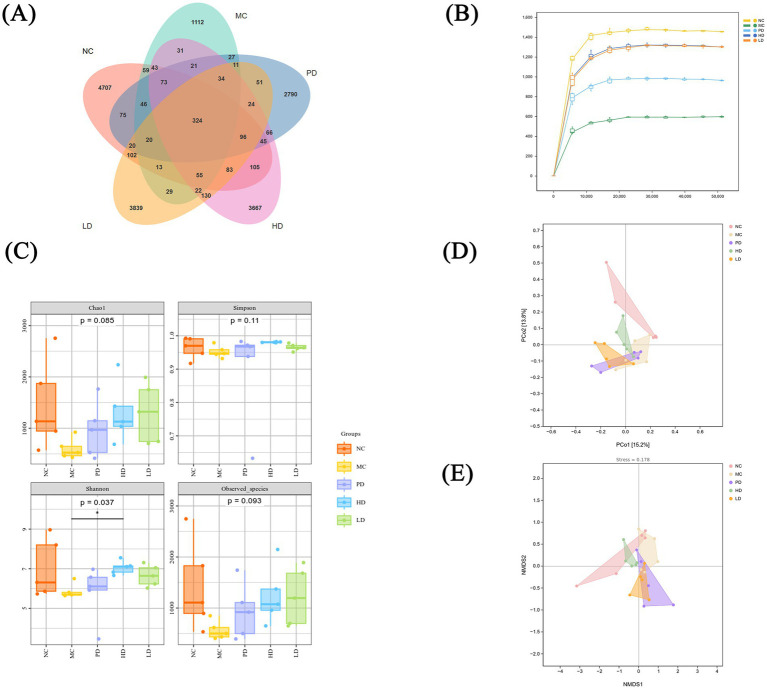
Analysis of the gut microbiota in mice with loperamide hydrochloride-induced constipation and MRZZ intervention (*n* = 5). **(A)** OTU Venn diagram of five groups of mouse feces. **(B)** *α*-diversity sparse curves. **(C)** α-diversity box plots based on the Chao 1, Simpson, Shannon, and Observed_species indices. **(D)** PCoA of *β*-diversity. **(E)** NMDS of β diversity.

The gut microbiota is crucial for the human body, helping to protect the enteric nervous system and restore intestinal function (Li et al., 2023). We analyzed 25 fecal samples from 5 groups of mice using 16S rRNA sequencing. 5,866, 1920, 3,723, 4,819, and 4,853 operational taxonomic units (OTUs) were identified in the NC, MC, PD, HD, and LD groups, respectively, with 324 common OTUs identified (Figure 5A). The HD and LD groups showed more OTUs than the MC group, indicating that MRZZ increased the number of species within the treated groups.

The samples were used to determine the α-diversity index based on OTUs with ≥ 97% sequence identity. The α-diversity sparse curve shown in Figure 5B indicates that the curve trend tended to flatten with the increasing depth of the stretching level. The species diversities of the LD and HD of MRZZ were the closest to that of the NC group, followed by those of the PD group and the MC group, suggesting that MRZZ increased the diversity of the bacterial flora in the intestinal tract of mice. Diversity was quantified using the Chao1, Simpson, Shannon, and Observed_species indices to evaluate the diversity and richness of the gut microbiota in each mice group (Figure 5C). The species richness and diversity indices performed well, with the LD and HD groups performing well; the Shannon indices differed significantly between the groups (*p* < 0.05). Although the Chao1 and Observed_species indices did not reveal significant differences between groups (*p* > 0.05), the index values of the MC group were lower than those of the NC group, and the MRZZ intervention increased the species abundance and diversity.

*β*-diversity was targeted for comparative analysis of microbiota among the different groups; therefore, the samples were analyzed via principal coordinates analysis (PCoA) and non-metric multidimensional scaling (NMDS) analysis, based on OTU species richness information. PCoA (Figure 5D) and NMDS (Figure 5E) analyses showed relative separation between the different groups, indicating that all five groups had distinct floral structures. In addition, the sampling points of the HD group were closer to those of the NC group, suggesting that MRZZ treatment may have reduced community structure differences. These findings suggest that MRZZ improves the species diversity of the gut microbiota in constipated mice, potentially by regulating the gut microbiota and improving the intestinal microecological environment, thereby alleviating constipation.

### 3.7 Changes in gut microbiota caused by MRZZ

**Figure 6:**
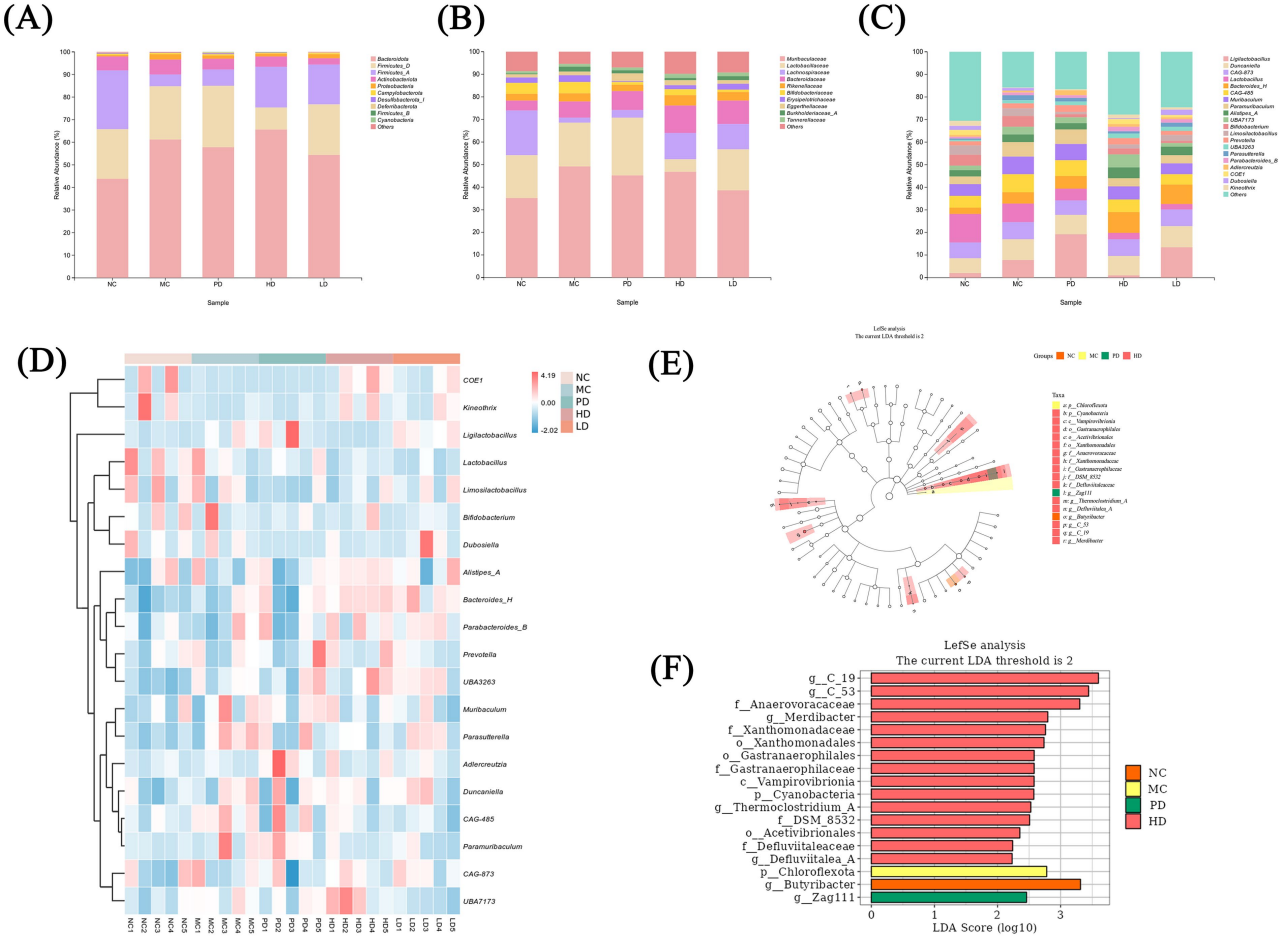
Analysis of the gut microbiota of mice with loperamide hydrochloride-induced constipation and MRZZ intervention (*n* = 5). **(A)** Bar graph showing the composition of the 10 most abundant species in different groups at the phylum level. **(B)** Bar graph showing the 10 most abundant species in different groups at the family level. **(C)** Bar graph showing the 20 most abundant species at the genus level in different groups. **(D)** Heat map of the 20 most abundant microbial species at the genus level. **(E)** Taxonomic-branching plot of LEfSe. **(F)** LDA bar chart of LEfSe.

The gut microbiota was tested in different groups, and the taxonomic characterization of the bacterial community was performed at different taxonomic levels (Figure 6). At the phylum level, the dominant phyla in all groups were *Bacteroidota* and *Firmicutes*, followed by *Actinobacteriota*, *Proteobacteria*, and others (Figure 6A). Compared with the NC group, the MC group had higher relative abundances of *Bacteroidetes*, *Actinobacteria*, and *Proteobacteria* and a decreased proportion of *Firmicutes*. Previous findings have shown that the proportion of *Bacteroidota* is higher than normal in patients with constipation, whereas that of *Firmicutes* is lower (Guo et al., 2020), which is consistent with the present results. In contrast, MRZZ treatment reversed this trend in the mice model, restoring the abundance of *Bacteroidota* and *Firmicutes* to levels similar to those in the NC group.

At the family level (Figure 6B), the HD and LD groups showed lower relative abundances of *Muribaculaceae* and *Lactobacillaceae* and higher proportions of *Lachnospiraceae* than did the MC group. Relative abundances (Figure 6C) and thermograms (Figure 6D) of the top 20 microbiota were determined at the genus level to further assess their distribution. MRZZ consumption increased the abundance of *Duncaniella*, *Bacteroides_H*, *Alistipes_A*, *UBA3263*, and *Parabacteroides_B* and decreased that of *CAG-873*, *Lactobacillus*, *CAG-485*, *Paramuribaculum*, *Prevotella*, and *Muribaculum*. These results indicate that MRZZ restored the composition and structure of the gut microbiota at different levels.

Differences in community composition across groups were compared using the linear discriminant analysis (LDA) effect size, and species with thresholds of > 2 were represented using taxonomic-branching diagrams (Figure 6E) and LDA histograms (Figure 6F). The NC, MC, and PD groups each had 1 taxon with a significant difference in abundance, whereas the HD group had 15 taxa with significant differences in abundance. In the NC group, *g_Butyribacter* was the most abundant bacterial genus, whereas *p_Chloroflexota* was more abundant in the MC group. In the PD group, the abundance of *g_Zag111* was significantly elevated. In the HD group, the abundances of *p_Cyanobacteria*, *c_Vampirovibrionia*, and *o_Gastranaerophilales* were significantly higher than those in the other groups. Taxonomic-branching diagrams revealed different degrees of abundance changes in the gut microbiota of each group.

### 3.8 Correlation of gut microbiota with significant differentially expressed genes

**Figure 7:**
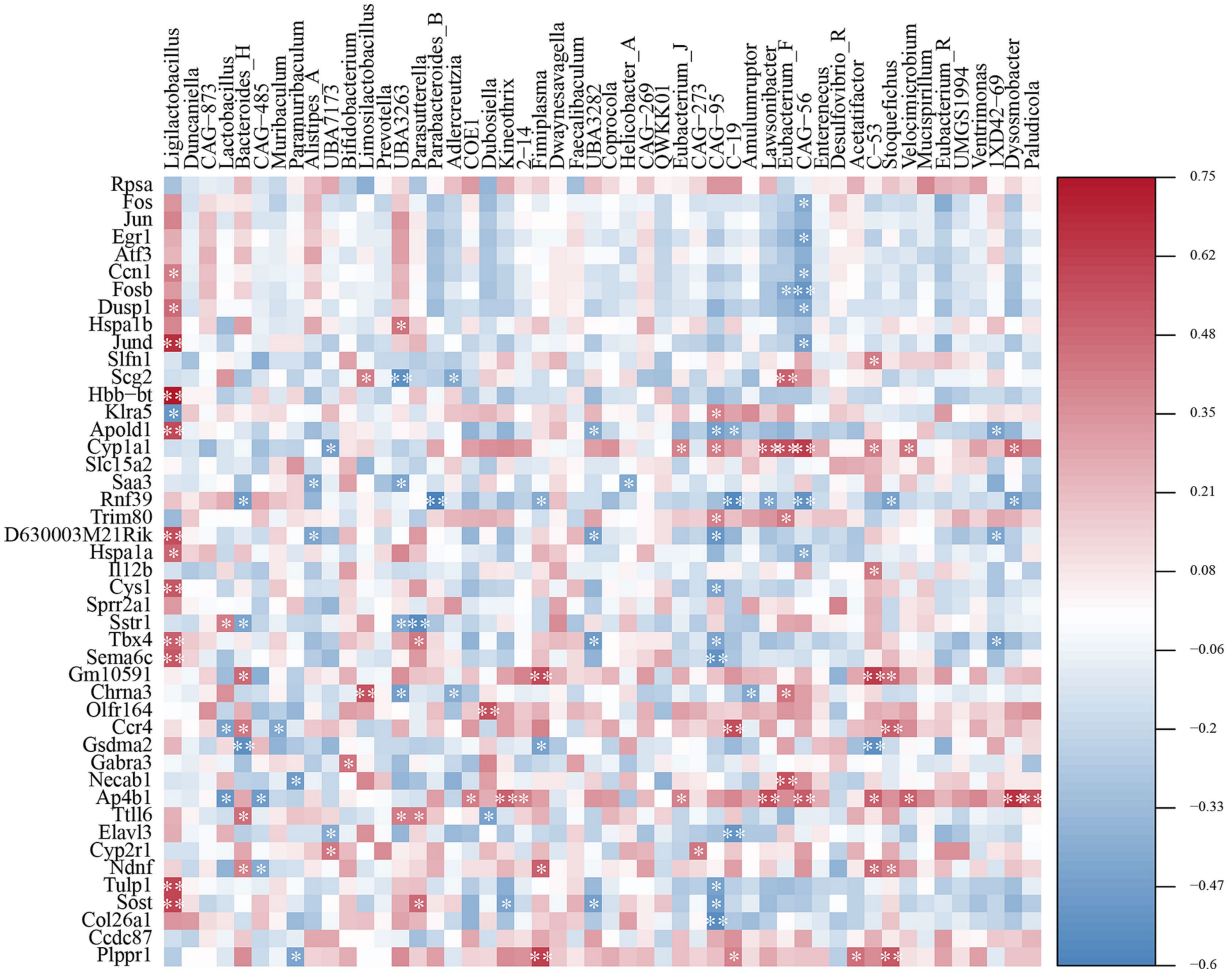
Spearman correlation analysis of the correlations between specific genera and differentially expressed genes. Positive correlations (> 0) are shown in red, with correlation, and negative correlations (< 0) are shown in blue. Shading indicates the strength of each correlation and statistical significance: *p < 0.05, **p < 0.01.

Correlations between the gut microbiota and transcriptomics were assessed using Spearman’s correlation analysis (Figure 7). The abundance of *Ligilactobacillus* was significantly positively correlated with differentially expressed genes such as *Ccn1, Dusp1, Jund, Hbb-bt, Apold1, D630003M21Rik, Hspala, Cys1, Tbx4, Sema6c, Tulp1,* and *Sost*, and negatively correlated with *Klra5*. The abundance of *Lactobacillus* was strongly positively correlated with *Sstr1* and negatively correlated with *Ccr4* and *Ap4b1* expression. The abundance of *Bacteroides* was significantly negatively correlated with *Rnf39, Sstr1,* and *Gsdma2* and was significantly positively correlated with *Gm10591, Ccr4, Ttll6,* and *Ndnf*.

## 4 Discussion

This study used tiger nut oil as a new carrier and integrated four Chinese herbs with homology of medicine and food—*Cannabis sativa* L., *Rehmannia glutinosa* Libosch., *Citrus aurantium* L., and *Atractylodes macrocephala* Koidz.—to develop MRZZ as a health food product. According to the theory of TCM, food therapy should be chosen on a priority instead of drugs to treat certain mild nonfunctional diseases. Compound formulas, a distinctive feature of TCM, often demonstrate greater therapeutic efficacy than single-component treatments due to the combined use of multiple components (Wang et al., 2022). In the preliminary stage, we used ultra-high-performance liquid chromatography-mass spectrometry (UPLC-MS) to identify 59 chemical components in MRZZ (Li et al., 2024). In addition, this study selected the Chinese patent medicine Xiangdanqing capsules as a positive control drug. The main ingredient of this formulation is *Astragalus membranaceus* Fisch. (Huangqi), *Aloe barbadensis* Miller (Luhui), and *Ginkgo biloba* L. (Baiguo), which are Chinese herbs with homology between medicine and food. It has a similar composition to that of MRZZ and relieves constipation. This study aimed to investigate the alleviating effect of MRZZ on loperamide hydrochloride-induced constipation in mice. Our results showed that MRZZ treatment improved the symptoms of constipation in mice. This improvement may be attributed to the regulation of the gut microbiota in mice, increased levels of SCFAs and serum gastrointestinal regulatory peptides, and the suppression of inflammatory responses.

The effects of MRZZ were verified by determining fecal water content, time to the first black stool, and gastrointestinal transit rate as primary measures of constipation in mice before and after MRZZ consumption (Li et al., 2014). Figure 1 indicates that MRZZ consumption improved relevant parameters, increased the efficiency of gastrointestinal transit (*p* < 0.001), reduced the time to the first black stool (*p* < 0.001), and increased the fecal water content (*p* < 0.001). We observed that the HD group gained weight more slowly than the other groups. We speculate this may be due to the intestinal lubrication effect of MRZZ, which promotes gastrointestinal motility and increases bowel movement frequency.

The results of the histological analyses are shown in Figure 2. H&E staining indicated that intestinal epithelial cells in the MC group were damaged and that the intestinal tissue structure was abnormal, whereas the MRZZ-treated mice exhibited a healthy colonic microvillus morphology, and the intestinal cell inflammatory damage was significantly repaired. Immunofluorescence staining further showed that the MRZZ group had higher fluorescence intensities of ZO-1 and Occludin. The upregulation of tight junction proteins enhances intestinal barrier integrity and maintains structural stability, which may play a protective role in cells (Hader et al., 2023; Sayoc-Becerra et al., 2020). Moreover, serum levels of gastrointestinal-related regulatory peptides are associated with intestinal motility. GAS regulates the degree of pyloric sphincter relaxation and promotes gastrointestinal motility (Li et al., 2023). MTL, which is densely distributed in the small intestine, promotes electrolyte transport and protease secretion in the gastrointestinal tract and is secreted at lower levels in patients with constipation (Huang et al., 2022). SP is involved in cell proliferation and immune regulation, which can stimulate interstitial calcium stromal cells to inhibit the development of intestinal injury (Jiang et al., 2020). Therefore, SP, MTL, and GAS can act as excitatory neurotransmitters that enhance gastrointestinal motility and promote defecation. In this study, monitoring gastrointestinal-related regulatory peptides indicated that MRZZ administration increased the serum levels of SP, MTL, and GAS in constipated mice (*p* < 0.05, Figures 3A–C), suggesting that MRZZ helped to normalize intestinal function and may play a crucial role in alleviating constipation.

SCFAs are metabolites of indigestible carbohydrates in the human body formed by the gut microbiota and include acetic, propionic, and butyric (Zhu et al., 2025). MRZZ consumption restored the fecal SCFA content, with the HD group showing significantly higher levels of acetic, propionic, butyric, and isobutyric acids (Figures 3D–H). Increased acetic acid production can lead to increased fecal water content, promote colonic motility, and increase the gastrointestinal transit rate (Wang et al., 2020), consistent with our findings related to fecal water content. Butyric acid helps to maintain gastrointestinal health by regulating intestinal epithelial cells and limiting intestinal inflammation, and is beneficial in treating constipation (Hodgkinson et al., 2023; Zhuang et al., 2019). Propionic and valeric acids can stimulate intestinal peristalsis, promote colonic propulsive motility, act on smooth muscle cells, and regulate ion channel activity to improve constipation (Rondeau et al., 2003), consistent with our results related to the first black stool and gastrointestinal transit rate. SCFAs can promote intestinal peristalsis and help maintain the physiological morphology of the intestinal cells (Parada Venegas et al., 2019). The increase in SCFA content further explains the intact intestinal mucosal structure observed via H&E staining in the MRZZ group, suggesting that SCFAs may be involved in MRZZ’s potential constipation-relieving mechanism.

However, further research is needed to verify the effectiveness of MRZZ in treating constipation. Besides altering the levels of regulatory neurotransmitters in the gastrointestinal tract, high and low levels of inflammatory factors may lead to changes in the intestinal barrier (Zhang et al., 2021). The NOD-like receptor signaling pathway regulates inflammatory responses and serves as a potential therapeutic target for gastrointestinal diseases (Zhou et al., 2023). *Jund* expression was significantly lower in the HD group than in the MC group. As a key factor in cellular regulation, *Jund* downregulation leads to changes in intestinal permeability (Zhang et al., 2023), which may help to alleviate constipation. *Slfn1* regulates intestinal epithelial differentiation in response to intestinal pathological conditions (Vomhof-DeKrey et al., 2021). Its upregulation in the HD group is speculated to possibly protect the intestinal barrier. Cytochrome P450 (CYP) enzymes play important roles in cellular metabolism and homeostasis as membrane-bound heme proteins whose efficacy is maintained despite their involvement in drug metabolism to treat constipation (Zhao et al., 2021). Simultaneously, *Cyp1a1*, a member of the cytochrome P450 family, was significantly upregulated in the HD group, and its regulatory mechanism was involved in intestinal homeostasis and repair (Foerster et al., 2022). These findings suggest that altered genes and related pathways may play key roles in alleviating loperamide hydrochloride-induced constipation.

Gut microbiota influences host health and is closely related to the causes of constipation (Lai et al., 2023). Previous data suggest that constipation can be alleviated by regulating the levels of gut microbiota (Liu et al., 2021). Therefore, we analyzed the potential mechanism by which MRZZ relieves constipation in terms of the gut microbiota. The *α*-diversity in the MRZZ groups was higher among the bacterial communities, compared with the MC group (Figures 5B,C), and was closer to that of the control group, which may reflect the fact that more communities in the host body correspond with a healthier environment.

At the phylum level (Figure 6A), the core composition of the gut microbiota in mice was dominated by *Firmicutes* and *Bacteroidetes*. *Firmicutes* contain a high number of beneficial bacteria with good resistance and are the main source of butyric acid among the SCFAs (Browne et al., 2021). MRZZ treatment altered the predominant bacterial species in mice, increasing the abundance of *Firmicutes* compared to the MC group, while also increasing the butyric acid content. In addition, *Proteobacteria* are potential pro-inflammatory bacteria that include many pathogenic genera (Sheng et al., 2019). Administration of MRZZ contributed to a relative decrease in the abundance of *Proteobacteria*, which may signal the restoration of intestinal homeostasis.

During constipation, an intestinal microecological imbalance leads to a decrease in the abundance of beneficial microbiota and an elevated ratio of conditionally pathogenic to potentially pathogenic bacteria. *Lactobacillus* and *Bifidobacterium* are often regarded as probiotics that can alleviate constipation (Tang et al., 2022). However, studies have shown that *Lactobacillus* competes with SCFA-producing bacteria, such as *Prevotella*, for substrates, which not only reduces the abundance of other SCFA-producing microbial communities but also inhibits the synthesis of SCFAs, such as butyric acid (Montgomery et al., 2024). This is consistent with our findings, where both the abundance of *Prevotella* and SCFAs levels increased after MRZZ treatment, potentially due to the reduced abundance of *Lactobacillus*. In addition, linoleic and linolenic acids, which are rich in *Cannabis sativa* L., are metabolized by the gut microbiota to fatty acids, further affecting the lipid bilayer in the bacterial cell membrane and the bacterial population (Druart et al., 2014; Kim et al., 2000). Our 16S rRNA sequencing analysis showed that MRZZ treatment altered the intestinal microbial structure in mice with loperamide hydrochloride-induced constipation. In addition, the correlation analysis revealed that *Ligilactobacillus* was positively correlated with *Jund* expression (*p* < 0.01), and *Ligilactobacillus* was more abundant in the MC group—consistent with the results of the previously mentioned *Jund* gene regulation (Figure 7). *Ccr4* regulates cells by binding to G-protein-coupled receptors (Ou et al., 2016). *Ccr4* was significantly and synergistically correlated with *Bacteroides* anomalies, which can promote the expression of gut-associated proteins and increase the production of acetic acid, further promoting colonic motility (Hooper et al., 2001; Yamada et al., 2021). Spearman’s correlation analysis revealed significant associations between the transcriptome and gut microbiota.

Although this study shows that MRZZ emulsion can relieve constipation, its mechanism of action requires further exploration. For example, the intervention effect of MRZZ under the depletion model can be further studied by adding tiger nut oils and excipients alone as control groups, or a sterile model can be established to transplant the fecal microbiota of mice treated with MRZZ to further verify its mechanism of action. In addition, MRZZ contains a variety of Chinese herbs, and identifying effective active ingredients can provide new insights into the process of relieving constipation using emulsions. We will continue conducting human consumption experiments and safety re-evaluations.

## 5 Conclusion

**Figure 8:**
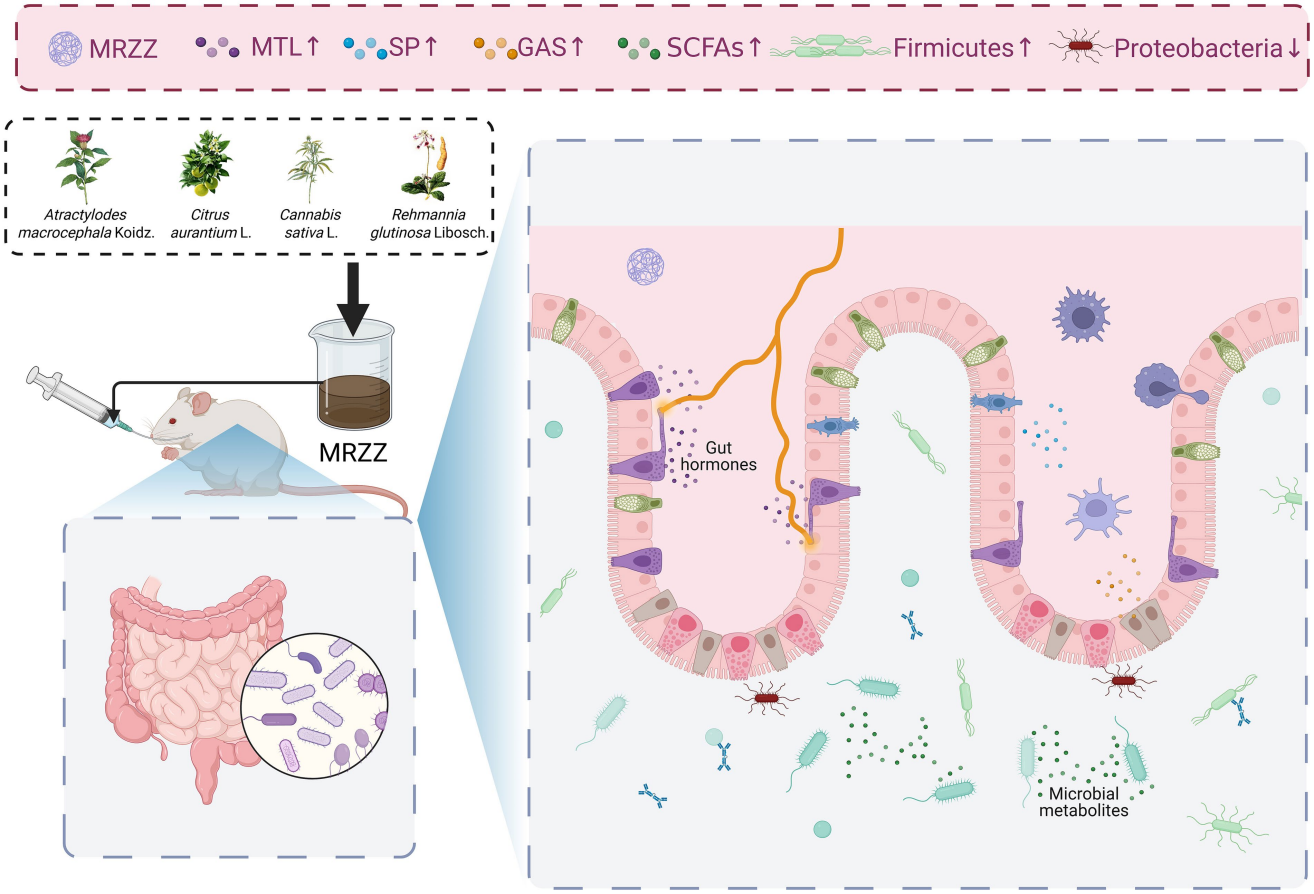
Potential mechanism of action of MRZZ in alleviating loperamide-induced constipation in mice.

This study indicates that MRZZ can effectively relieve constipation and enhance intestinal function. The potential mechanism (Figure 8) may involve increasing the levels of relevant gastrointestinal regulatory neurotransmitters, such as MTL, SP, and GAS, as well as SCFAs (especially acetic acid and isobutyric acid). Simultaneously, MRZZ may synergistically enhance its relief effect on constipation by inhibiting intestinal inflammatory responses and regulating the composition of the gut microbiota—such as increasing the relative abundance of *Firmicutes* and reducing potentially pathogenic bacteria such as *Proteobacteria*. In conclusion, as a functional food, MRZZ may enhance the application of TCM in the treatment of constipation. However, further research is needed to elucidate the detailed mechanisms by which MRZZ alleviates constipation.

## Data Availability

The raw data presented in the study have been deposited to National Center for Biotechnology Information (NCBI) under the BioProject number PRJNA1300621 and PRJNA1300799.

## Footnotes

^1^ https://www.Genescloud.cn/home
